# Supplementary material for: Heparin-based hydrogel scaffolding alters the transcriptomic profile and increases the chemoresistance of MDA-MB-231 triple-negative breast cancer cells
Source: Biomater Sci. 2020 Feb 13;8(10):2786–96. doi: 10.1039/c9bm01481k (PMC7497406; doi:10.1039/c9bm01481k)
Supplement: Supplementary file 2 [file BM-008-C9BM01481K-s002.zip › Supplementary File 4/HP-B hydrogel + EGF (s)vControl/Pathways/my_analysis.Gsea.1545201170987/HALLMARK_PEROXISOME.html]

Details for gene set HALLMARK\_PEROXISOME[GSEA]

|  || Dataset | expr.class.cls#HYDEGF\_versus\_CONTROL.class.cls#HYDEGF\_versus\_CONTROL\_repos |
| Phenotype | class.cls#HYDEGF\_versus\_CONTROL\_repos |
| Upregulated in class | CONTROL |
| GeneSet | HALLMARK\_PEROXISOME |
| Enrichment Score (ES) | -0.44762436 |
| Normalized Enrichment Score (NES) | -1.9076177 |
| Nominal p-value | 0.0 |
| FDR q-value | 6.2713126E-4 |
| FWER p-Value | 0.005 |
Table: GSEA Results Summary

  

Fig 1: Enrichment plot: HALLMARK\_PEROXISOME      
 Profile of the Running ES Score & Positions of GeneSet Members on the Rank Ordered List

  

| PROBE | DESCRIPTION (from dataset) | GENE SYMBOL | GENE\_TITLE | RANK IN GENE LIST | RANK METRIC SCORE | RUNNING ES | CORE ENRICHMENT || 1 | ERCC3 | na |  |  | 582 | 2.035 | -0.0044 | No |
| 2 | CLN8 | na |  |  | 879 | 1.766 | 0.0027 | No |
| 3 | ACAA1 | na |  |  | 1222 | 1.561 | 0.0048 | No |
| 4 | ABCC5 | na |  |  | 1401 | 1.467 | 0.0143 | No |
| 5 | NUDT19 | na |  |  | 1460 | 1.446 | 0.0298 | No |
| 6 | SLC25A19 | na |  |  | 1594 | 1.396 | 0.0407 | No |
| 7 | HRAS | na |  |  | 2033 | 1.257 | 0.0338 | No |
| 8 | MLYCD | na |  |  | 2419 | 1.147 | 0.0284 | No |
| 9 | SLC23A2 | na |  |  | 2699 | 1.081 | 0.0276 | No |
| 10 | CTBP1 | na |  |  | 2892 | 1.029 | 0.0308 | No |
| 11 | PEX6 | na |  |  | 3525 | 0.900 | 0.0092 | No |
| 12 | CLN6 | na |  |  | 4163 | 0.788 | -0.0140 | No |
| 13 | GSTK1 | na |  |  | 4536 | 0.729 | -0.0241 | No |
| 14 | ABCB4 | na |  |  | 4791 | 0.689 | -0.0286 | No |
| 15 | DHCR24 | na |  |  | 4964 | 0.657 | -0.0292 | No |
| 16 | CEL | na |  |  | 5266 | 0.611 | -0.0371 | No |
| 17 | PEX5 | na |  |  | 6261 | 0.468 | -0.0831 | No |
| 18 | CADM1 | na |  |  | 7418 | 0.314 | -0.1396 | No |
| 19 | SLC25A17 | na |  |  | 7642 | 0.284 | -0.1476 | No |
| 20 | DLG4 | na |  |  | 7838 | 0.257 | -0.1546 | No |
| 21 | ESR2 | na |  |  | 8227 | 0.219 | -0.1721 | No |
| 22 | LONP2 | na |  |  | 9141 | 0.110 | -0.2184 | No |
| 23 | ABCD1 | na |  |  | 9269 | 0.094 | -0.2239 | No |
| 24 | HSD11B2 | na |  |  | 9679 | 0.052 | -0.2446 | No |
| 25 | ABCB9 | na |  |  | 9837 | 0.031 | -0.2524 | No |
| 26 | ACOT8 | na |  |  | 10148 | -0.002 | -0.2686 | No |
| 27 | SIAH1 | na |  |  | 10385 | -0.033 | -0.2805 | No |
| 28 | CRAT | na |  |  | 10668 | -0.062 | -0.2945 | No |
| 29 | ERCC1 | na |  |  | 10709 | -0.065 | -0.2957 | No |
| 30 | ACSL1 | na |  |  | 11022 | -0.109 | -0.3107 | No |
| 31 | HSD3B7 | na |  |  | 11035 | -0.112 | -0.3099 | No |
| 32 | YWHAH | na |  |  | 11375 | -0.143 | -0.3258 | No |
| 33 | ITGB1BP1 | na |  |  | 11555 | -0.169 | -0.3330 | No |
| 34 | STS | na |  |  | 11556 | -0.169 | -0.3308 | No |
| 35 | PEX11B | na |  |  | 12133 | -0.234 | -0.3579 | No |
| 36 | ATXN1 | na |  |  | 12145 | -0.236 | -0.3555 | No |
| 37 | ABCD3 | na |  |  | 12429 | -0.275 | -0.3668 | No |
| 38 | PEX11A | na |  |  | 12605 | -0.299 | -0.3721 | No |
| 39 | RETSAT | na |  |  | 12706 | -0.315 | -0.3733 | No |
| 40 | TOP2A | na |  |  | 12732 | -0.319 | -0.3705 | No |
| 41 | NR1I2 | na |  |  | 12945 | -0.335 | -0.3773 | No |
| 42 | CRABP2 | na |  |  | 13038 | -0.346 | -0.3777 | No |
| 43 | MVP | na |  |  | 13105 | -0.354 | -0.3766 | No |
| 44 | MSH2 | na |  |  | 13118 | -0.356 | -0.3727 | No |
| 45 | PEX14 | na |  |  | 13302 | -0.386 | -0.3774 | No |
| 46 | FDPS | na |  |  | 13646 | -0.433 | -0.3898 | No |
| 47 | IDH2 | na |  |  | 14028 | -0.492 | -0.4034 | No |
| 48 | ECH1 | na |  |  | 14216 | -0.507 | -0.4067 | No |
| 49 | CNBP | na |  |  | 14420 | -0.541 | -0.4104 | No |
| 50 | GNPAT | na |  |  | 14869 | -0.614 | -0.4260 | No |
| 51 | SCP2 | na |  |  | 15058 | -0.644 | -0.4276 | No |
| 52 | ACSL4 | na |  |  | 15387 | -0.701 | -0.4357 | No |
| 53 | ACOX1 | na |  |  | 15588 | -0.740 | -0.4367 | No |
| 54 | IDE | na |  |  | 15687 | -0.759 | -0.4321 | No |
| 55 | ECI2 | na |  |  | 15984 | -0.815 | -0.4372 | Yes |
| 56 | SMARCC1 | na |  |  | 15991 | -0.816 | -0.4271 | Yes |
| 57 | PEX2 | na |  |  | 16073 | -0.838 | -0.4206 | Yes |
| 58 | SLC25A4 | na |  |  | 16080 | -0.840 | -0.4101 | Yes |
| 59 | TSPO | na |  |  | 16294 | -0.896 | -0.4098 | Yes |
| 60 | SLC35B2 | na |  |  | 16353 | -0.911 | -0.4012 | Yes |
| 61 | ALDH9A1 | na |  |  | 16400 | -0.923 | -0.3918 | Yes |
| 62 | PEX13 | na |  |  | 16767 | -1.016 | -0.3979 | Yes |
| 63 | FADS1 | na |  |  | 16789 | -1.024 | -0.3859 | Yes |
| 64 | FABP6 | na |  |  | 17155 | -1.128 | -0.3906 | Yes |
| 65 | SEMA3C | na |  |  | 17409 | -1.218 | -0.3882 | Yes |
| 66 | ACSL5 | na |  |  | 17434 | -1.228 | -0.3738 | Yes |
| 67 | ELOVL5 | na |  |  | 17447 | -1.235 | -0.3586 | Yes |
| 68 | HMGCL | na |  |  | 17468 | -1.244 | -0.3437 | Yes |
| 69 | BCL10 | na |  |  | 17922 | -1.452 | -0.3488 | Yes |
| 70 | EHHADH | na |  |  | 17931 | -1.456 | -0.3306 | Yes |
| 71 | ISOC1 | na |  |  | 17943 | -1.462 | -0.3125 | Yes |
| 72 | FIS1 | na |  |  | 17956 | -1.465 | -0.2943 | Yes |
| 73 | PRDX1 | na |  |  | 18026 | -1.499 | -0.2788 | Yes |
| 74 | IDI1 | na |  |  | 18098 | -1.547 | -0.2627 | Yes |
| 75 | DHRS3 | na |  |  | 18222 | -1.612 | -0.2485 | Yes |
| 76 | VPS4B | na |  |  | 18613 | -1.943 | -0.2440 | Yes |
| 77 | HSD17B11 | na |  |  | 18643 | -1.977 | -0.2202 | Yes |
| 78 | CAT | na |  |  | 18668 | -2.014 | -0.1957 | Yes |
| 79 | IDH1 | na |  |  | 18690 | -2.035 | -0.1707 | Yes |
| 80 | RDH11 | na |  |  | 18715 | -2.072 | -0.1455 | Yes |
| 81 | CDK7 | na |  |  | 18889 | -2.336 | -0.1246 | Yes |
| 82 | SOD1 | na |  |  | 18910 | -2.370 | -0.0953 | Yes |
| 83 | SOD2 | na |  |  | 18995 | -2.569 | -0.0668 | Yes |
| 84 | HSD17B4 | na |  |  | 19012 | -2.605 | -0.0343 | Yes |
| 85 | PRDX5 | na |  |  | 19161 | -3.438 | 0.0019 | Yes |
Table: GSEA details [plain text format]

  

Fig 2: HALLMARK\_PEROXISOME      
 Blue-Pink O' Gram in the Space of the Analyzed GeneSet

  

Fig 3: HALLMARK\_PEROXISOME: Random ES distribution      
 Gene set null distribution of ES for **HALLMARK\_PEROXISOME**
